# Markovian and Non-Markovian Protein Sequence Evolution: Aggregated Markov Process Models

**DOI:** 10.1016/j.jmb.2011.06.005

**Published:** 2011-08-26

**Authors:** Carolin Kosiol, Nick Goldman

**Affiliations:** 1 Institut für Populationsgenetik, Vetmeduni Vienna, Veterinärplatz 1, A-1210 Wien, Austria; 2 European Bioinformatics Institute, EMBL Outstation-Hinxton, Wellcome Trust Genome Campus, Hinxton, Cambridge CB10 1SD, UK

**Keywords:** protein evolution, amino acid substitution models, codon models, aggregated Markov models, rate heterogeneity, AMP, aggregated Markov process, MRCA, most recent common ancestor, HMM, dden Markov model

## Abstract

Over the years, there have been claims that evolution proceeds according to systematically different processes over different timescales and that protein evolution behaves in a non-Markovian manner. On the other hand, Markov models are fundamental to many applications in evolutionary studies. Apparent non-Markovian or time-dependent behavior has been attributed to influence of the genetic code at short timescales and dominance of physicochemical properties of the amino acids at long timescales. However, any long time period is simply the accumulation of many short time periods, and it remains unclear why evolution should appear to act systematically differently across the range of timescales studied. We show that the observed time-dependent behavior can be explained qualitatively by modeling protein sequence evolution as an aggregated Markov process (AMP): a time-homogeneous Markovian substitution model observed only at the level of the amino acids encoded by the protein-coding DNA sequence. The study of AMPs sheds new light on the relationship between amino acid-level and codon-level models of sequence evolution, and our results suggest that protein evolution should be modeled at the codon level rather than using amino acid substitution models.

## Highlights

► Markov models are essential to many applications in evolutionary studies of proteins. ► We introduce AMPs to model protein sequence evolution. ► We use AMPs to study the relationship between codon and amino acid evolutions. ► Our results explain previous claims of non-Markovian behavior of protein sequences. ► We suggest that codon models may be preferred to amino acid models.

## Introduction

In 1968, Dayhoff *et al.* introduced a first model of protein sequence evolution, resulting in the development of the widely used amino acid replacement matrices known as the PAM matrices.^1^ Since Dayhoff's PAM matrices, there have been increasingly good descriptions of the average patterns and processes of evolution of large collections of protein sequences, as well as more and more specialized matrices considering functional and structural properties of proteins.^2–5^ Such models are widely used in comparative sequence analyses.^6,7^

Mathematically speaking, all these models are time-homogenous Markov models defined by the assumption that each amino acid evolves independently of time and of its past history. The instantaneous rate matrix, which represents the patterns of the substitution process and specifies the model completely, is the same at any time in a time-homogenous model.^6^ In fact, if the rate matrix can be written as the product of a scalar function of time and a constant matrix, that is, *Q*(*t*) = *r*(*t*)*Q*, then *r*(*t*) can be interpreted as an overall rate of evolution varying over time and *Q* can be interpreted as a constant pattern of amino acid replacements. In this case, the overall evolutionary rate and time are confounded,^8,9^ and over any time period [*t*_0_,*t*_1_], the process so defined cannot be distinguished from that defined by the time-homogenous $Q\left(t\right)=\overset{―}{r}Q$, where $\overset{―}{r}$ is the mean rate in that period, equal to $\int_{t_{0}}^{t_{1}}r\left(t\right)dt/\left(t_{1}-t_{0}\right)$, if it is only observed at *t*_0_ and *t*_1_.^10^ In this paper, we refer to Markov processes defined by instantaneous rate matrices that can be written in the form *r*(*t*)*Q* as time-homogeneous. While not precisely accurate, it is a convenient shorthand for a class of Markov processes that cannot be distinguished from time-homogeneous ones using the available data.

For sequence evolution on a phylogenetic tree, imagine that, after a speciation (or gene duplication) event, a pair of sequences evolves from their common ancestor according to a time-homogeneous Markov model. After some time, we may measure the differences and the divergence level between the two sequences, and because the model is time homogeneous, the sequences will continue to evolve according to the same process, leading to more differences and higher divergence levels. This model implies that the patterns of substitutions taking place are the same at low and high sequence divergences. Even if the overall rate of evolution varies between lineages (i.e., the instantaneous rate matrix varies by a constant multiplicative factor) or over time within lineages, a properly implemented inference procedure is able to infer the constant patterns of evolutionary changes.^11^ However, while most work in phylogenetic modeling has concentrated on devising improved Markov models, some criticisms have been directed at the models' time-homogeneous and Markov natures themselves.

Henikoff and Henikoff derived a series of BLOSUM matrices, which are probability matrices but are not based on a Markov model.^12^ They counted all the amino acid replacements between conserved subblocks of aligned protein sequences from many different protein families in the BLOCKS database. The subblocks were made by single-linkage clustering about a percentage identity threshold, and different matrices were obtained by varying this threshold. The matrices of the BLOSUM series are identified by a number after the matrix (e.g., BLOSUM62), which refers to the percentage identity of the subblocks of multiple aligned amino acids used to construct the matrix. Although these percentages indicate different divergence levels between the aligned proteins that give rise to each matrix, there is no assumption of common patterns of amino acid change over evolutionary time: the BLOSUM matrices are not based on an evolutionary model, and it is not possible to generate the BLOSUM matrix series simply by interpolating or extrapolating.

BLOSUM matrices often perform better than PAM matrices for the purpose of amino acid sequence alignment or database searching.^12^ This may be because protein sequences behave in a non-time-homogeneous or non-Markov manner, a hypothesis that could have serious consequences for the fields of maximum likelihood and Bayesian phylogenetics, which are based on time-homogeneous Markov models.

Mitchison and Durbin tried to find one global and constant instantaneous rate matrix *Q* that could generate, as an exponential family (see below), a series of protein replacement matrices they had estimated empirically (also from BLOCKS).^13^ This would have been a time-homogeneous Markov process explanation of their observations, but they could not find a *Q* that gave a good fit. Furthermore, Benner *et al.* (hereafter referred to as BCG) inferred protein replacement matrices from sets of sequences separated by different divergence levels and found qualitative differences in the substitution patterns.^14^ They concluded that the evolutionary process changed as a function of sequence divergence, that the assumption that high divergence can be modeled by extrapolating the patterns of low sequence divergence does not hold and that amino acid sequence evolution is non-Markovian.

### Thought experiments that expose the fallacy that evolution is different depending on when it is observed

**Fig. 1:**
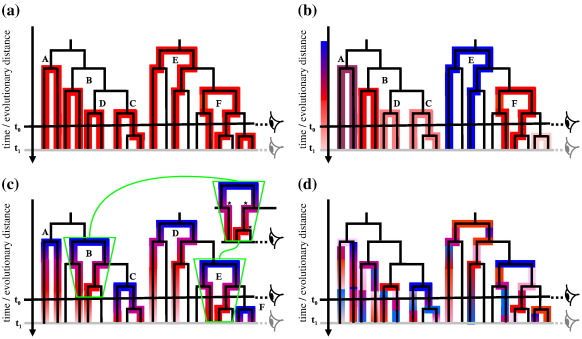
Observing evolutionary processes at different time points. Throughout the figure, different colors represent different substitution processes along a branch, and we consider observing different sequence pairs (colored lineages) at time *t*_0_ (black eye) and at later time *t*_1_ (gray eye). (a) Simple time-homogeneous Markov model. (b) Replacement dynamics dependent on time since MRCA, color coded according to spectrum to the left of panel. (c) Approach of Benner *et al.*: replacement dynamics dependent on speciation and duplication events and subsequent elapsed time. The “callout” (green box) with asterisks indicates lineage splits that are not observed when pairwise comparisons B and E are used. (d) Modeling the “average” process using a time-homogenous approach: process changes without trend are indicated by random change in color patterns. Note that, for all the processes illustrated in (a)–(d), we assume that corrections for the occurrence of multiple substitutions have been made so that the “snapshot” we take at different times should not lead to different observations caused by multiple substitutions.

Figure 1a illustrates the assumption made by time-homogeneous Markov models. All patterns of change are constant over all evolutionary time, represented by one shade of red along the branches of the phylogenies that feature in any sequence comparison (colored branches are sampled; uncolored branches are not). The “eyes” on the right and the associated horizontal lines indicate what would be observed at two different time points (denoted *t*_0_ and *t*_1_). For this simple model, the process observed back in time to the ancestor is the same from any time point (e.g., black eye at *t*_0_ cf. gray eye at *t*_1_) and regardless of which sequences are compared (comparisons A–F). BCG's finding that amino acid evolutionary patterns appear different depending on the divergence level of proteins compared implies that such a simple time-homogeneous Markov assumption cannot be correct.

BCG formulated perhaps the most detailed criticism of standard Markov models. Although they observed different patterns of replacement for different divergence levels, their explicit rejection of Markovian evolution^14^ is unfounded, as they did not explore the possibility of time-dependent Markov processes or the extension of the state space to recover the Markov property.^15^ Nevertheless, as so much current phylogenetic theory relies on time-homogeneous Markov processes, it is important to see if such models can be reconciled with BCG's and Mitchison and Durbin's observations.

Invoking different evolutionary models for every different sequence is of little interest: it is only the general applicability of a particular model that makes it widely useful. Figure1b represents one simple possible explanation of replacement patterns being different for different divergence levels. This model is time-homogeneous Markov but has a different pattern of replacements according to the time of their most recent common ancestor (MRCA). This is illustrated using shades from blue to violet for high divergence levels (Fig. 1b; e.g., comparisons A and E), bright red for medium divergence levels (B and F) and pale-red shades for low divergence levels (C and D), as observed at the level of the black eye (*t*_0_). The time axis is in effect linked with a color scale distinguishing different evolutionary processes for different divergence levels before the present. This interpretation is, however, problematic for two reasons. First, notice that the comparison labeled A observed at *t*_0_ represents a level of divergence that is different from that of comparison B observed at the same time, as indicated by their different colors, but imagine time elapsing until *t*_1_, the level of the gray eye. Comparison B is now equivalent to the earlier observation A, yet the pattern of changes observed in A (relative to the black eye) and B (gray eye) do not match. This thought experiment regarding observation points that differ in time shows that this model cannot give rise to observed patterns of changes characterized by the time since sequences' evolutionary divergence. The same argument can be made by contrasting, for example, comparison B made at *t*_0_ and comparison D made at *t*_1_.

Second, still referring to Fig. 1b, we highlight the point that the evolutionary histories relating a pair of sequences do not correspond unambiguously to one divergence at a unique position in evolutionary time. Imagine that, in Fig. 1b, the two trees represent the evolution of the same set of sequences, but with different pairwise comparisons highlighted. Different choices of comparisons (e.g., A and B in the left-hand tree and E in the right-hand tree) include sequences with common history yet different evolutionary pattern because the pairwise divergence is greater. This is inconsistent, further illustrating that sequences' evolutionary histories are not uniquely associated with one specific divergence level. The same point is made by considering comparisons F, C and D. BCG's observations cannot be consistent with evolutionary dynamics that are constant over time (Fig. 1a and b).

A more sophisticated interpretation of BCG's conclusions can remove some of the inconsistencies highlighted in Fig. 1b by having the evolutionary process change over time. If all protein sequences evolved in a concerted fashion, each undergoing identical substitution dynamics at the same point in actual (clock) time and with those dynamics varying over time, then inferred patterns of change could be consistent with BCG's observations. Such a model could be Markov (though clearly not time-homogeneous), but this level of synchronization of evolutionary dynamics is, however, entirely unrealistic. A more plausible argument would be that patterns of change could alter at points in the tree where lineages split (e.g., duplication can create gene copies free from the same functional constraints as their ancestors).^16^ BCG's conclusion was that such a scenario, with protein evolution transitioning from domination by the genetic code soon after divergences to domination by amino acids' physicochemical properties at greater distances, could account for their observations. This hypothesis is represented in Fig. 1c as Markov evolution dependent on time since the last lineage split. If evolutionary patterns depend only on the time since the MRCA of the sequences compared (Fig. 1c, A–F), then evolutionary dynamics would seem different depending on the compared sequences' divergence levels. The time of observation (e.g., *t*_0_ or *t*_1_) does not alter this finding or lead to any inconsistency.

However, only a small proportion of BCG's pairwise comparisons will have gene duplication (as opposed to speciation) events as their MRCA. Only those few that do are likely to have altered evolutionary patterns,^17^ and even these will generally have been subject to these altered evolutionary patterns only for a short time near to the duplication event.^18^ Further, an explanation such as this also assumes duplications to occur only at the MRCA of each observed pairwise comparison. However, there is no guarantee that there have been no subsequent lineage splitting events after the MRCA of an observed pair of sequences. Indeed, this too is an unlikely scenario: often the case will be as illustrated by comparisons B–E in Fig. 1c, which contain multiple lineage splits that are not observed (for B and E, highlighted by asterisks in the “callout” region of the figure) in addition to the one at the MRCA that is. Consequently, it is not tenable to invoke an explanation of the observation of time-dependent protein evolution based on actual duplication/speciation events, since there is no distinction in BCG's data between sequence pairs that are true sister groups and those separated by intermediate (unanalyzed or simply unobserved) descendants of the same common ancestor.

In contrast, Fig. 1d illustrates our understanding of the complexity of evolutionary dynamics assessed over a large collection of related and unrelated proteins. There are various different processes (colors) in different lineages; there are both gradual changes at different rates and abrupt changes, and these may or may not coincide with lineage splits. The position of changes in evolutionary process is largely random with respect to the position in the actual underlying or observed trees and is not coordinated from one lineage to another. In this case, the end result of observations taken at any time and of sequences of any level of divergence will be the mixture of many processes. While each may be Markov and time-homogeneous, the overall effect may be highly time-inhomogeneous on a per-lineage basis. However, estimated over large assemblages of protein examples, the average inferred evolutionary dynamics may remain the same, with no biases induced by what sequences are observed, which pairs are compared or when our experiment takes place.

This series of thought experiments indicates that, contrary to BCG's suggestion, in the most realistic case, the “average” process should be time homogeneous and should be the same if estimated from enough sequences, irrespective of their divergence levels and irrespective of whether it is estimated in 1994, in 2011 or in one million or one hundred million years time. Long periods of evolution are no more than the accumulation of many short periods of evolution, unaware of when they will be observed.

### Alternative explanations of experimental results

The time-homogeneous approach is logically consistent where BCG's explanation is not, but the simple time-homogeneous models considered above are unable to explain the experimental results of Benner *et al.*^14^ and Mitchison and Durbin^13^ and the success of the BLOSUM matrices.^12^ Explanations involving processes that are nonhomogeneous in time, relying (logically inconsistently) on specific (implausible) duplication and speciation events or invoking complex switches of substitution dynamics have also failed to explain BCG's observations. Accepting those observations but not necessarily their authors' conclusions, we investigate other factors that could have caused the differences in inferred evolutionary dynamics depending on observed divergence levels.

Markov processes are very successful at modeling the average behavior of collections of chance events. Trying to retain a time-homogeneous Markov framework while performing this investigation, in this paper, we use aggregated Markov processes (AMPs)^19^ to model protein evolution as Markovian at the DNA (codon) level but observed (via the genetic code) only at the amino acid level. All previous studies of non-Markovian behavior were on the amino acid level only and often with inference techniques that are less advanced than those now available.^20^ Evolution, however, occurs at the DNA level. Furthermore, codon-level models have been tested and improved^21,22^ so that we can hope that we have adequate codon models to base this study on. We show that many time-inhomogeneous findings for protein evolution can be explained by time-homogeneous Markov models of the evolution of codon sequences that are observed at the level of amino acids.

## Theory

### Time-homogeneous Markov models for sequence evolution

The time-homogeneous Markov model asserts that one protein sequence is derived from another by a series of independent mutations, each changing one character in the ancestral sequence to another character in its descendant during evolution. We consider only models that assume independence of evolution at different sites. A continuous-time Markov process is defined by its *N* × *N* instantaneous rate matrix *Q* = (*Q*_*ij*_)_*i*__,*j* = 1,…*N*_, where *N* is the number of character states. Two types of character alphabets will be considered for protein evolution here: amino acids (*N* = 20) and codons (*N* = 61, if stop codons are discarded). For *i* ≠ *j*, the matrix entry *Q*_*ij*_ represents the instantaneous rate of change from state *i* to state *j*, independently at each site. Our assumption of time-homogeneity means that *Q*_*ij*_ are constant in time (or that any time dependence is through a scalar rate factor is described in Introduction).

Changes at each site occur as a Poisson process with these given rates while waiting in a particular state. The total rate at which any change from that state occurs is the sum of all the rates of changes from that state, and this determines the waiting time in a given state before moving to another. The *Q*_*ii*_ entry of the matrix is set to be minus the sum of all other entries in that row, representing (− 1 times) the rate at which changes leave state *i*:

$$Q_{ii}=-\sum_{j\neq i}^{N}Q_{ij}$$

Molecular sequence data consist of observed character states at some given time, and the quantity most commonly needed for calculations is the probability of observing a given character after evolutionary time *t* ≥ 0 has elapsed. We denote by *P*_*ij*_(*t*) the probability of a site being in state *j* after time *t*, given that the process started in state *i* at that site at time 0. We can write the probabilities *P*_*ij*_(*t*) as an *N* × *N* matrix that we denote *P*(*t*) and that is determined via the relationship^23^

(1) $$P(t)={\text{e}}^{tQ}$$

where the exponential of a matrix is defined by the following power series, with *I* being the appropriate identity matrix:

(2) $${\text{e}}^{tQ}=I+tQ+\frac{{\left(tQ\right)}^{2}}{2!}+\frac{{\left(tQ\right)}^{3}}{3!}+\cdots$$

In practice, this power series is calculated numerically using standard linear algebra techniques.^24^ The most popular method in molecular phylogeny uses eigen-decomposition.^6^ A series *P*(*t*_1_), *P*(*t*_2_), … that can be derived in this way from the same instantaneous rate matrix *Q* is referred to as an exponential family of matrices.

If a Markov process is left evolving for a long time, the probability of finding it in a given state converges to a value independent of the starting state; this distribution is known as the equilibrium distribution π = (π_1_,…,π_*N*_). The equilibrium distribution π can be found by solving π*P*(*t*) = π for any *t* > 0 or equivalently^23^ π*Q* = 0.

Time (*t*) and rate (*Q*_*ij*_) are confounded, and without extrinsic information, only their product can be inferred.^8,9^ Consequently, we can normalize the instantaneous rate matrix with any factor. Typically in phylogenetic applications, *Q* is normalized so that the mean rate of replacement at equilibrium (∑_*i*_∑_*j*__ ≠ *i*_π*_i_Q_ij_*) is 1, which means that times (evolutionary distances) are measured in units of expected substitutions per site.

### Amino acid models

For the amino acid substitution models in this paper, we assume that amino acid sites in an alignment evolve independently according to the same reversible Markov process defined by a 20 × 20 instantaneous rate matrix. Dayhoff *et al.* introduced the first amino acid model in the form of a substitution probability matrix. However, an instantaneous rate matrix *Q* can easily be calculated from such a probability matrix.^11^ In this study, we use the Dayhoff model provided in the phylogenetic software package PAML;^25^ other common models give qualitatively similar results. Following common practice, we often refer to the “PAM distance”. This effectively corresponds to 100× the expected number of amino acid replacements per amino acid site.^11^

### Codon models

Markov models of codon substitution were first proposed by Goldman and Yang^21^ and Muse and Gaut.^26^ In this paper, we mainly refer to the model M0 from Yang *et al.*^22^ For this model, the elements of *Q* are defined as:

(3) $$Q_{ij,i\neq j}=\{\begin{array}{ll} \text{0} & \text{if }i\text{ or }j\text{ is a stop codon or}\\ & \text{the change }i\to j\text{ requires > 1 nucleotide substitution}\\ \pi_{j} & \text{if }i\to j\text{ is a synonymous transversion}\\ \pi_{j}\kappa & \text{if }i\to j\text{ is a synonymous transition}\\ \pi_{j}\omega & \text{if }i\to j\text{ is a nonsynonymous transversion}\\ \pi_{j}\kappa \omega & \text{if }i\to j\text{ is a nonsynonymous transition} \end{array}$$

where the parameter κ is the transition/transversion rate ratio, ω is the nonsynonymous/synonymous rate ratio and π_*j*_ is the equilibrium frequency for each codon *j*. Because of the interpretation of the parameter ω as a bias toward (ω > 1) or away from (ω < 1) nonsynonymous changes, this model and its variants are widely used in the detection of natural selection.^22,27,28^ For amino acid models and codon models, variation of rates among sites in proteins has been modeled. Often, a discretized Gamma distribution of rates is considered,^29^ and we use this and similar approaches below.

As we often discuss amino acid-level and codon-level processes together, we help distinguish these contexts by using subscripts *i*, *j*,… for codons and *x*, *y*,… for amino acids.

### Aggregated Markov processes

Protein sequence evolution of amino acids, as well as of codons, has been modeled using Markov processes. However, the evidence found against time-homogeneous Markov models has all derived from amino acid-level analyses of protein sequences, whereas protein evolution occurs at the level of coding DNA. Here, we consider a model that is Markov on the underlying codon level, but which we interpret as its corresponding amino acids.

Suppose, for example, that between times *t*_*k*_ and *t*_*k*__ + __1_, the following substitution has occurred in a coding region:

**Figure:**
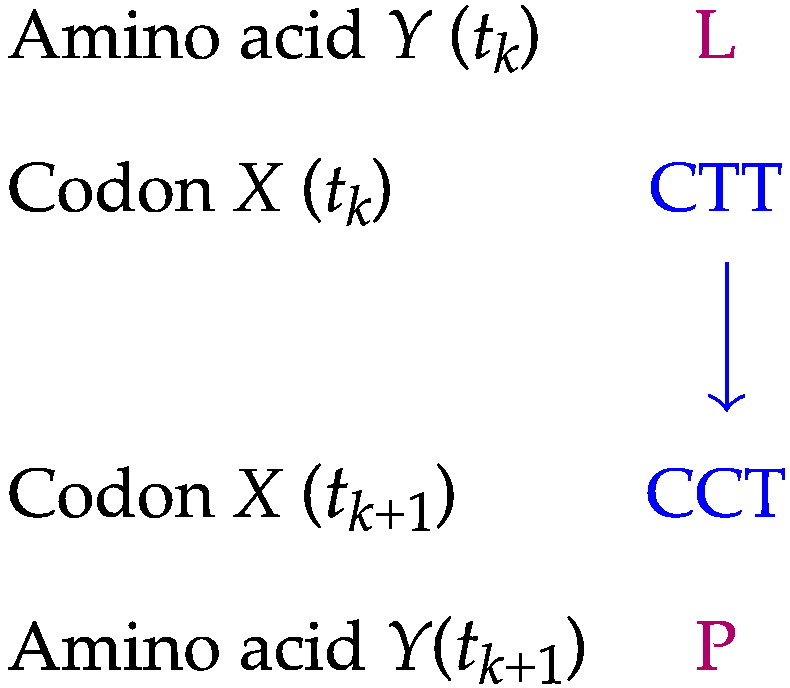

We assume that such substitutions are generated by a continuous-time time-homogeneous Markov process {*X*(*t*), *t* ≥ 0} on the codon level with state space *C* = {AAA,AAC,…,TTG,TTT} and equilibrium distribution π and probability matrices *P*(*t*) = e^*Qt*^ as above. Further, we suppose that the codons are not directly observable but that a deterministic function of the underlying Markov process [i.e., *Y*(*t*) = *f*(*X*(*t*)), where *f* maps the state space *C* to the aggregate set *A* = {A,R,N,…,V}], can be observed. Clearly, we consider observing the amino acids encoded by the codons, with *f* defined by the universal genetic code. The observable process of amino acids {*Y*(*t*), *t* ≥ 0} is then called an AMP.^19^ The dependence structure for the site highlighted in the example above is represented by the following graph:

**Figure:**
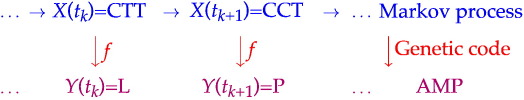

Given only the amino acid-level observations *Y*(*t*), it is impossible to tell whether the substitution of leucine (L) with proline (P) was caused by a substitution from CTT to CCT, from CTC to CCC, from CTA to CCA or from CTG to CCG. [We assume only single-nucleotide changes; a more general model might permit double- and triple-nucleotide changes instantaneously (e.g., CTT → CCA), which results in a larger set of codon substitutions compatible with each amino acid replacement.^30^] Consequently, the probability of a change to proline (P) does depend not only on the present amino acid leucine (L) but also on the hidden state *X*(*t*_*k*_). The stochastic process *Y*(*t*) describing the amino acid evolution is therefore non-Markovian.

More formally, AMPs are a subclass of hidden Markov models (HMMs); HMMs also allow the observed *Y*(*t*) to be probabilistically determined given *X*(*t*). The theory of HMMs says that the stochastic process *X*(*t*) on the state space is Markov but that the observable process *Y*(*t*) is non-Markov.^31^ It is therefore clear that a Markov process of codon evolution will lead to non-Markovian observations of amino acid sequence evolution. Below, we ask whether this can explain the time-dependent observations that other authors have recorded.

Yang *et al.* have described another way of deriving an amino acid substitution model from a codon model.^32^ However, they constrain the amino acid model to be Markovian, fixing the rates of amino acid changes equal to the total rates of all corresponding codon changes. Being Markovian by construction, such models cannot explain observations of non-Markovian amino acid substitution.

### Log-odds matrices and BCG's experiments

Rather than compare inferred instantaneous rate matrices *Q*, BCG illustrated their findings by discussing elements of the log-odds matrices (*L*) often used as scoring matrices in database search and alignment programs. Positive scores in a log-odds matrix designate a pair of residues that replace each other more often than expected by chance; negative scores designate pairs that replace each other less than would be expected by chance. Log-odds matrices are related to probability matrices *P*(*t*) (see below), and similar to them, they depend on an evolutionary distance or time *t*. To make meaningful comparisons of log-odds matrices derived from sequences with different divergence levels, one needs to carry out some normalization, and BCG achieved this by computing matrices standardized to *t* = 2.5 (250 PAM). Below, we adopt BCG's procedures to estimate probability matrices, calculate log-odds matrices and normalize them.

BCG split 1.7 million pairwise aligned amino acid sequences from the MIPS database^33^ into 10 sets based on bands of divergence levels (4.7–6.4, 6.4–8.7, 8.7–11.8, 11.8–16, 16–22, 22–29, 29–40, 40–54, 54–74 and 74–100 PAMs). Denoting the average PAM distance in each set by *t*_*k*_ for *k* = 1,…,10, for each *k*, they compiled a matrix of counts *T*(*t*_*k*_), where *T*_*xy*_(*t*_*k*_) is the number of substitutions from amino acid *x* to amino acid *y* observed in a given set of sequences, and a diagonal matrix *N*(*t*_*k*_), with *N*_*xx*_(*t*_*k*_) the total observed number of amino acids of type *x*. Since, from a pairwise alignment, it is not possible to decide whether a substitution is from *x* to *y* or from *y* to *x*, half of each substitution is counted in one direction and half is counted in the other. For each of the PAM bands, BCG then estimated amino acid substitution matrices using the formula

$$P(t_{k})=T(t_{k})\times {\left[N(t_{k})\right]}^{-1}$$

These matrices are each extrapolated to a divergence of 1 PAM (0.01 expected substitutions per site):

(4) $$P(1\text{ PAM})=P(t=0.01)={\left[P(t_{k})\right]}^{1/t_{k}}={\left[T(t_{k})\times {\left[N(t_{k})\right]}^{-1}\right]}^{1/t_{k}}$$

and converted to a 250-PAM (*t* = 2.5) log-odds matrix:

(5) $$L_{xy}(250)=10log_{10}\frac{P_{xy}(2.50)}{f_{y}}=10log_{10}\frac{{\left[{\left(P(0.01)\right)}^{250}\right]}_{xy}}{f_{y}}\;\text{for all }x\neq y$$

where *f*_*y*_ = *N*_*yy*_(*t*_*k*_)/∑*_z_N_zz_* (*t*_*k*_) is the frequency of amino acid *y* in each data set.

**Fig. 2:**
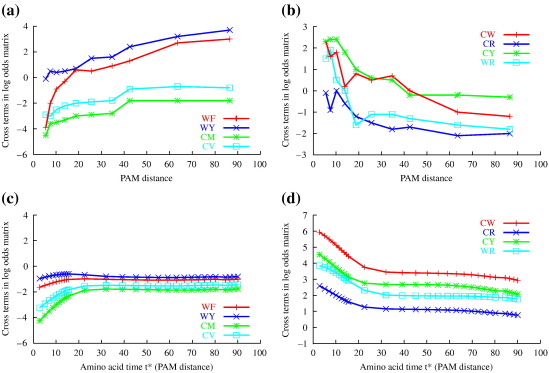
Graphs of some cross terms of *L*(250) log-odds matrices. For example, the line labeled WF represents the value of the cross term (off-diagonal element) [*L*(250)]_WF_ computed from data at divergence levels between 0 and 100 PAMs. (a and b) Graphs redrawn from Benner *et al.*,^14^ colored for clarity. (c and d) Results of simulations under an AMP model.

BCG illustrated their results by plotting values of *L*_*xy*_(250) for various amino acids *x* and *y*. Their results are reproduced in Fig. 2a and b. These clearly indicate that certain elements of the log-odds matrices vary as a function of the PAM distance of the sequences from which they were estimated, behavior that is inconsistent with time-homogeneous Markovian evolution. Looking for a biological explanation for these findings, BCG proposed an interpretation that the genetic code influences protein evolution strongly at early stages of divergence, while physicochemical properties are dominant at later stages. For example, they inferred from the values of *L*_CW_(250) (Fig. 2b) that substitutions from cysteine (C) to tryptophan (W) are frequent at small PAM distances because only a single base change is necessary (TGC or TGT to TGG), whereas at larger PAM distances, these substitutions are infrequent because the side chain of tryptophan (W) is large and hydrophobic while the side chain of cysteine (C) is small and can form disulfide bonds inaccessible to tryptophan (W). Similar arguments were made for other amino acid substitutions illustrated in Fig. 2a and b. However, as explained in our thought experiments above, it does not make sense to base such explanations on divergence levels or speciation events.

### Exponential families and Mitchison and Durbin's experiments

**Fig. 3:**
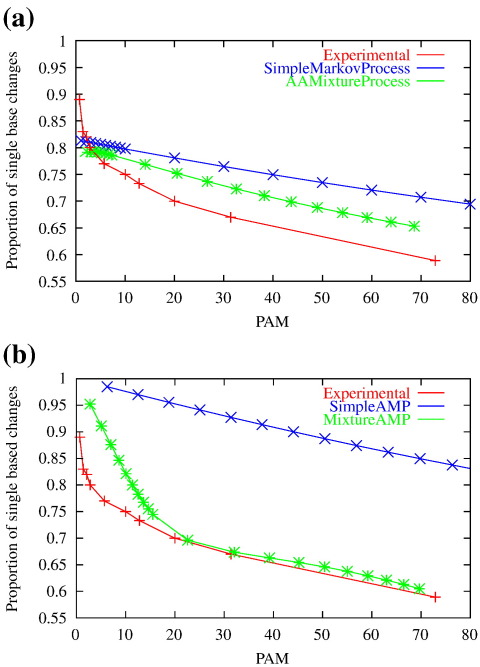
Comparison to Mitchison and Durbin's results. (a) Experimental data, simple time-homogeneous Markovian and mixture of time-homogeneous Markov processes on the amino acid level. (b) Experimental data and aggregated processes of a codon-level time-homogeneous Markov process both with and without rate heterogeneity.

Mitchison and Durbin estimated amino acid substitution probability matrices from experimental data using maximum likelihood methods,^13^ inferring 10 matrices from multiple alignments taken from the same BLOCKS database used to derive the BLOSUM matrices.^12^ They then tried to identify an exponential family that would generate the series of matrices (i.e., explain their observations as a time-homogeneous Markov process) but were unable to do so. However, they provided interesting analysis and diagnostics giving insight into the reasons why their approach failed. In their diagnostics, they considered the proportion of amino acid changes that may be explained by a single-nucleotide change and the way this proportion changes over time. To compute this, they summed substitution probability matrix entries over all amino acid substitutions that can be achieved via a single-nucleotide change and took the ratio of this to the probability of any change:

(6) $$\frac{\sum_{(x\to y)\in \Delta_{1}}\;P_{xy}(t)}{\sum_{x}\;\sum_{y\neq x}\;P_{xy}(t)}$$

where Δ_1_ is the set of amino acid changes requiring only a single-nucleotide change. This value is plotted in Fig. 3a (“Experimental”) and shows an initial rapid decline followed by a slower decline for more distant protein comparisons. This is suggestive of a change in evolutionary dynamics that is not consistent with the near-linear decrease observed for time-homogeneous Markovian amino acid sequence evolution (Fig. 3a, “SimpleMarkovProcess”; see also below). As with BCG, naïve interpretation of these results again suggests that different processes are observed at different timescales. Since our thought experiments indicate that sequence evolution cannot be different depending simply on when we make our observations, this finding is another that we hope to explain via AMPs.

### Simulation methods

Simulation of evolving sequences as a way of testing hypotheses and evaluating the idealized behavior of evolutionary models is well established.^34^ We use simulated codon data to investigate if aggregation (AMPs) can lead to observations similar to those of BCG and Mitchison and Durbin. Working at the codon level, we calculated $P(t_{k}^{*})={\text{e}}^{Qt_{k}^{*}}$ (see above) using values of *t*_*k*_^⁎^ covering a range similar to that used by BCG. The frequency of observing codon *i* in one sequence and *j* in another is then given by π*_i_P_ij_*(*t*_*k*_^⁎^). Letting *C*_*x*_ and *C*_*y*_ represent the sets of codons that code for amino acids *x* and *y*, respectively, we simulate aggregated (amino acid level) data by noting that the frequency of observing amino acid *x* in one sequence and amino acid *y* in the other sequence is then

(7) $$\sum_{i\in C_{x}}\;\sum_{j\in C_{y}}\;\pi_{i}P_{ij}(t_{k}^{*})$$

To create AMP substitution matrices from this simulation data, we apply the methods of BCG to these idealized data and set

$$T_{xy}(t_{k}^{*})=\sum_{i\in C_{x}}\;\sum_{j\in C_{y}}\;\pi_{i}P_{ij}(t_{k}^{*})\;\text{and}\;N_{xx}(t_{k}^{*})=\sum_{y}\;T_{xy}(t_{k}^{*})$$

The matrices *T*(*t*_*k*_^⁎^) and *N*(*t*_*k*_^⁎^) are subjected to the same analyses performed by BCG and Mitchison and Durbin in order to see if AMPs can give an explanation of those authors' observations.

Furthermore, we do not use the prespecified codon times *t*_*k*_^⁎^ to normalize the matrices to time *t* = 0.01 [Eq. (4)] but, instead, base this normalization on inferred amino acid times. BCG had to rely on PAM distances *t*_*k*_ estimated from observed amino acid sequences, and it is important that we mimic this because the amino acid time estimates *t*_*k*_ may be systematically and nonlinearly biased relative to the codon times *t*_*k*_^⁎^ (see also below). PAML^25^ can perform this estimation based on frequencies such as those computed from Eq. (7). We used this method to estimate *t*_*k*_, the divergence levels of the AMP amino acid data, for use in normalizing probability matrices according to Eq. (4).

## Results

### Understanding the behavior of AMPs: the Chapman–Kolmogorov equation

The Chapman–Kolmogorov equation gives the method of combining probabilities from substitution patterns observed at intermediate time steps into longer-term probabilities when the underlying process is Markovian.^35^ Adherence to the Chapman–Kolmogorov equation is a necessary and sufficient condition for a process to be Markov and guarantees the ability to interpolate and extrapolate probabilities from observations at different time points.

For example, suppose we are interested in *P*(*Y*(*t*_1_) = M|*Y*(*t*_0_) = C), the probability of a change from amino acid cysteine (C) at time *t*_0_ to methionine (M) at time *t*_1_. (We concentrate on cysteine–methionine changes here for simplicity, but the same results hold generally for any amino acid pair.) However, imagine an experiment designed in such a way that we have three observation times (*t*_0_, *t*_1_ and an intermediate time τ) and that the data allow us to determine the amino acid substitution probabilities for the period from *t*_0_ to *t*_1_ and also for the periods from *t*_0_ to τ and from τ to *t*_1_.

If the AMP were Markovian, we could relate the probabilities of the observations in the different time periods by applying the Chapman–Kolmogorov equation:^23^

(8) $$P(Y(t_{1})=\text{M}|Y(t_{0})=\text{C})=\sum_{x}\;P(Y(t_{1})=\text{M}|Y(\tau )=x)\times P(Y(\tau )=x|Y(t_{0})=\text{C})\text{ for any }\tau \in [t_{0},t_{1}]$$

where the summation is over all 20 amino acids that might be observed at intermediate time τ. Conversely, if the Chapman–Kolmogorov equation does not hold, we know that the examined process is behaving in a non-Markovian manner.^35^

In the context of our earlier thought experiments, this corresponds to estimating probability matrices at intermediate times and using these to calculate the substitution probabilities at a later time. However, we now show that assuming Markovian behavior for observations (e.g., amino acid changes) generated by an AMP can lead to substantial error in the estimation of substitution probabilities. Interestingly, the time of observation does matter for the AMP, whereas it is irrelevant for a simple Markovian process. This begins to establish that AMPs may be able to explain some earlier authors' observations of non-Markovian protein evolution.

For the AMP representing the case that we only observe amino acids, we calculate the right-hand side of the Chapman–Kolmogorov equation by using probabilities derived from Eq. (7) above.

On the underlying codon level, the process is Markov by construction (see above). However, to confirm adherence to the Chapman–Kolmogorov equation and to compare codon results with amino acid results, for our codon model, we apply the following calculations. For *Y*(*t*_0_), we use the equilibrium distribution for the codons of cysteine (C) defined by

$${\left[\upsilon_{0}\right]}_{i}=\{\begin{array}{ll} \frac{\pi_{i}}{\sum_{j\in C_{\text{C}}}\pi_{j}} & \text{if }i\in C_{\text{C}}\\ 0 & \text{otherwise} \end{array}$$

where *C*_C_ is the set of codons coding for amino acid C. We consider a codon initially in a state described by this distribution and evolving (according to the Markov codon model) over time. For example, at time τ, the state distribution is υ_0_*P*(τ − *t*_0_) and the probability to be in amino acid state *x* at time τ is given by:

(9) $$\begin{array}{cc} P(Y(\tau )x|Y(t_{0})=\text{C}) & =\sum_{i\in C_{x}}{\left[\upsilon_{0}P\left(\tau -t_{0}\right)\right]}_{i}=\sum_{i\in C_{x}}\sum_{j\in C}{\left[\upsilon_{0}\right]}_{j}P{\left(\tau -t_{0}\right)}_{ji}\\ & =\sum_{i\in C_{x}}\sum_{j\in C}{\left[\upsilon_{0}\right]}_{j}P\left(Y\left(\tau \right)=i|Y\left(t_{0}\right)=j\right)\\ & =\sum_{i\in C_{x}}\sum_{j\in C_{\text{C}}}{\left[\upsilon_{0}\right]}_{j}P\left(Y\left(\tau \right)=i|Y\left(t_{0}\right)=j\right)\\ & =\sum_{j\in C_{\text{C}}}{\left[\upsilon_{0}\right]}_{j}\sum_{i\in C_{x}}P\left(Y\left(\tau \right)=i|Y\left(t_{0}\right)=j\right) \end{array}$$

**Fig. 4:**
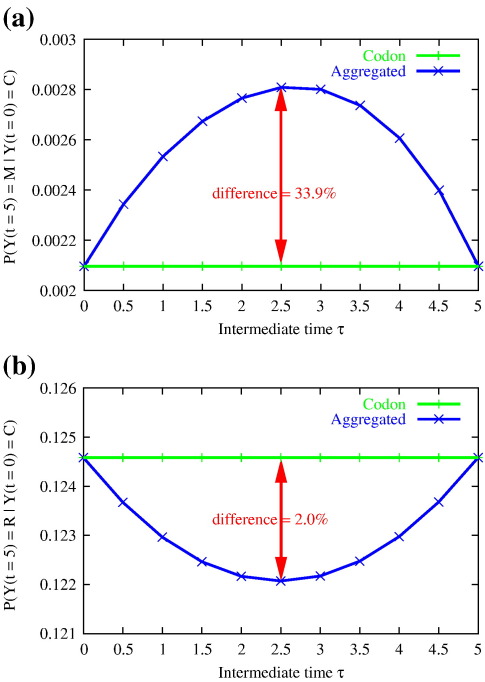
Dependence of transition probabilities on the time of intermediate observation. Transition probabilities for the purely Markov codon model (labeled Codon) and the AMP (Aggregated) are calculated using the right-hand side of the Chapman–Kolmogorov Eq. (8). (a) Probability of a change from cysteine (C) to methionine (M) via an intermediate step at time τ. Cysteine and methionine are distant in the genetic code (three-nucleotide changes). (b) Probability of a change from cysteine (C) to arginine (R) via an intermediate step at time τ. Cysteine and arginine are close in the genetic code (one-nucleotide change).

Using appropriate versions of Eq. (9), we have calculated the right-hand side of the Chapman–Kolmogorov Eq. (8) for *t*_0_ = 0, for *t*_1_ = 5 and for different intermediate times τ ∈ [0.0,5.0] for the purely Markov codon model and compare it to the simulated AMP results (only amino acids observed). We use a simple M0 model [Eq. (3)] with ω = 0.2 and κ = 2.5 and codon frequencies as specified in Supplementary Material. The results for the change from cysteine to methionine (C → M), as described above, and also for the change from cysteine to arginine (C → R) are shown in Fig. 4. Unlike the results from the Markovian codon process, the values derived from the AMP are not constant. In other words, the probabilities of amino acid substitution depend on the intermediate time τ when the amino acid sequences are observed. Considering similar plots for other amino acid substitutions (not shown), we note that this effect is particularly strong if the amino acids are distant in the genetic code (e.g., they are two- or three-nucleotide changes apart).

This confirms, therefore, that although the AMP is a time-homogeneous Markov process on the codon level, it is non-Markovian (and time dependent) when observations are aggregated to the level of amino acids. Perhaps the AMP can provide a logically consistent model that can explain BCG's claim that the time at which the evolutionary process is observed is relevant for the estimation of the substitution process.

### Comparison to BCG's results

The extrapolated matrices *L*(250) obtained from BCG's 10 data sets of different divergence levels should be the same if the underlying process of amino acid sequence evolution were time homogeneous. To check this, we used Dayhoff's amino acid model to simulate perfectly time-homogeneous Markov data in the form of pairwise alignments at different divergent levels *t*_*k*_ and applied BCG's inference procedure, as described above, to calculate the *L*(250) log-odds matrices. For these data, we confirmed that the elements of the log-odds matrix are not dependent on the divergence levels *t*_*k*_ (see Supplementary Material Fig. S1a and b) and, thus, that BCG's observations (Fig. 2a and b) are not consistent with time-homogeneous Markovian amino acid substitution.

Proteins often show variation in the rates of substitution at different protein sites. This can affect estimates of divergence levels^36^ and, thus, raises the question of whether rate heterogeneity at the amino acid level could have caused the time dependency effects BCG observed. Therefore, we also simulated data from a mixture process acting on amino acids, a methodology not available at the time of BCG's analysis, using a discretized Gamma distribution of rates of evolution over amino acid sites that is determined by the parameter α.^29^ To simulate a broad range of typical protein data, we used 182 values of α, representing an empirical distribution of values for typical globular proteins.^37^ However, although very slight changes in *L*(250) values could be observed, the effect was not as pronounced as that observed by Benner *et al.* (see Supplementary Material Fig. S1c and d). We conclude that although combined data from a mixture of time-homogeneous Markovian amino acid models with different values of α can generate observations with slight time dependence, a realistic distribution of α values cannot explain BCG's observations. In summary, we were unable to recreate observations of Benner *et al.* using time-homogeneous amino acid Markov models.

We next investigated whether simulations under AMP models could lead to observations similar to those of BCG. Initially, we used a simple M0 model (above), choosing realistic values of ω = 0.2 (moderate purifying selection), κ = 2.5 and codon frequencies as specified in Supplementary Material. The log-odds matrices showed some dependency on the PAM distance, but the magnitude of the effect on the log-odds elements did not reflect the strong variation of the log-odds of BCG's experimental data. Further trials with different parameter values led to qualitatively similar results (not shown).

However, introducing a more realistic model for among-site rate variation by using a Gamma model^29^ clearly compared better with Benner's plots from experimental data (not shown). Finally, instead of determining rate variation by a Gamma distribution, we illustrate the effect using rate categories specified “by hand”. Excellent results were achieved by aggregation of simulated data from a codon model with among-site rate variation defined by 12 relative rate categories:

(10) $$\begin{array}{cccc} r_{1}=0.000001 & r_{2}=0.00001 & r_{3}=0.0001 & r_{4}=0.001\\ r_{5}=0.01 & r_{6}=0.1 & r_{7}=0.15 & r_{8}=0.2\\ r_{9}=0.3 & r_{10}=0.5 & r_{11}=2.0 & r_{12}=8.738889 \end{array}$$

All categories were given the same probability (1/12), maintaining a mean rate of 1. While a Gamma distribution was not extreme enough, we note that our choice of distribution of evolutionary rates is still realistic. In particular, we needed very slowly evolving sites to explain the time dependence effect more than those given by a Gamma distribution; however, models allowing for a substantial proportion of invariant sites are widely used in phylogenetics.^38,39^

Results for this AMP model are shown in Fig. 2c and d and should be compared with Benner's results on experimental data in Fig. 2a and b. Similar to the graphs of the experimental data, the graphs of the simulated data show significant curvature. For the specific elements of the log-odds matrix that BCG plotted, the order and the trends of the graphs agree for the experimental and simulated AMP data. This shows that relatively complex but realistic time-homogeneous codon models can generate behavior similar to what BCG observed.

However, some ranges of the experimental graphs are different (Fig. 2). We speculate that this may reflect the fact that the M0 codon model is not fully realistic, for example, treating all synonymous changes and all nonsynonymous changes equally and assigning the same level of selective pressure (ω) to all protein sites. AMPs based on more complex parametric codon models^22^ or on empirical codon models^22^ might give a picture of the ranges of *L*(250) matrix values more in accord with BCG's empirical results. Also, at high PAM distances, the simulated graphs converge to zero as predicted by theory. In contrast, BCG's experimental graphs actually often cross the zero line, which we attribute to problems such as difficulty of aligning divergent sequences, noise, small sample sizes, and so on.

A final possibility not yet addressed is that BCG's experimental graphs could have the shapes they do not because of any systematic effect of molecular evolutionary processes but simply as a result of inferential noise. Our results from simulating data sets of approximately the sizes of those used by BCG indicate that the resulting levels of variability are not nearly sufficient to explain the difference in shapes between the curves shown in Fig. 2a and b (BCG) and c and d (our results) (see Supplementary Material Fig. S2 for details).

### Comparison to Mitchison and Durbin's results

We repeated Mitchison and Durbin's analysis using four simulations. We simulated data from Dayhoff's amino acid model as a simple time-homogeneous Markovian amino acid model and using a mixture of time-homogeneous Markovian amino acid models as described above, incorporating 182 rate heterogeneity (α) values.^37^ We also simulated data from an AMP based on the codon model described above, both without rate heterogeneity and with rate heterogeneity as given by the 12 rate categories in Eq. (10).

Figure 3 compares the results from these four models to the experimental data of Mitchison and Durbin. Figure 3a confirms that a simple time-homogeneous Markov model on the amino acid level does not fit their observations. Although the mixture of time-homogeneous amino acid models gives results somewhat closer to experimental data, it still predicts the proportion of single base changes to decrease fairly linearly. Thus, it appears that time-homogeneous Markovian amino acid process models alone cannot explain the observations of Mitchison and Durbin.

Figure 3b shows similar poor agreement between Mitchison and Durbin's results and the results from our AMP with no rate heterogeneity. However, there is much better agreement for our AMP incorporating rate heterogeneity. Although this combination of time-homogeneous codon model, rate heterogeneity and aggregation does not reflect precisely the behavior of the experimental results for small times (PAM distances), it does capture much better the rapid and nonlinear decline of the proportion of single base changes. Again, apparent differences in protein evolution on different timescales can in fact be explained by an AMP.

## Discussion

Since the work of Dayhoff *et al.*, there have been increasingly good empirical models of the average patterns and processes of evolution of large collections of amino acid sequence, as well as more and more specialized matrices considering functional and structural properties of proteins. However, while most work in phylogenetic modeling is aimed at devising improved time-homogeneous Markov models, some criticisms have been directed at the time-homogeneity assumption and the models' Markov nature itself. Studies on experimental protein sequence data (e.g., by Benner *et al.*^14^ and Mitchison and Durbin^13^) have observed different substitution patterns at different levels of sequence divergence. These observations indicate that amino acid sequence evolution behaves in a time-dependent manner.

While Benner *et al.* did not support their criticisms of the Markov nature of amino acid sequence evolution by consideration of time-dependent Markov processes, the claims that time-homogeneity was violated required further investigation.^14^ In a series of thought experiments, we have shown that past explanations (i.e., that the process of evolution is different for different divergence times) are irrational because the time of observation and the choice of sequences compared cannot have any influence on actual amino acid substitutions. However, time-homogeneous Markov models are fundamental to many applications in evolutionary studies, and we need to find some explanation for the observations.

We emphasize that our criticism of Benner *et al.*' interpretation of their results should not be taken to mean that we are arguing against the importance of the genetic code or of amino acids' physiochemical properties in evolutionary models. However, we argue that these influence the average substitution patterns observed over collections of proteins at all evolutionary distances in the same way. Indeed, studies of genome variation data have suggested an influence of physicochemical properties at the population level^40–42^ and, thus, within a far-shorter period than the distances discussed in this paper.

We have also shown that the time-dependent behavior described in the literature can be explained by modeling protein-coding DNA sequence evolution as an AMP that combines a time-homogeneous Markov model of codon evolution with rate heterogeneity among different codon sites of the protein and that evaluates what we would infer if we observed only encoded amino acid sequences at different divergence levels. This leads to a model that is non-Markovian on the observed amino acids, and we have focused on the consequences of non-Markovian behavior using the Chapman–Kolmogorov equation^35^ and comparisons to studies on experimental data by Benner *et al.*^14^ and Mitchison and Durbin.^13^ Although previous results^13,14^ cannot be explained by a pure time-homogeneous Markov model or a realistic mixture of such models on the amino acid level, the aggregated Markov model captures the qualitative behavior of empirical studies and leads to better agreement between models and empirical data. Although it does not incorporate any of the physicochemical properties considered by Benner *et al.* to be responsible for their results, the AMP in fact is able to capture quite accurately the form of the results interpreted by Benner *et al.* and Mitchison and Durbin as evidence of time-dependent evolution. We therefore conclude that the paradox that arose from past observations of time-dependent behavior can be resolved.

AMPs based on M0 (above) capture the high proportion of single base changes at very low divergence levels observed by Mitchison and Durbin because they assume that individual codon replacements involve only single bases [Eq. (3)].^13^ Studies that have investigated instantaneous occurrence of multiple base replacements suggest that these do arise in low numbers.^43,30^ AMPs based on models incorporating these events could lead to improved fit with Mitchison and Durbin's observations. We found that a high level of rate variation across sites was also needed to give a good fit to empirical results. An effect of this rate variation is to concentrate codon changes into a small number of highly variable sequence sites, leading to more changes per altered site and thus a higher proportion of altered sites requiring multiple base changes to explain observed amino acid differences. This contributes to the much steeper fall in the proportion of single base changes as divergence increases for the AMP with rate variation, as shown in Fig. 3b, and Fig. 2 illustrates the same effects at the level of individual amino acid replacements. An additional effect that contributes to the improved fit of our rate-heterogeneous AMP is systematic underestimation of divergence caused by model misspecification. Here, we estimate divergences from data generated by a rate-heterogeneous codon process, with an amino acid model assuming rate homogeneity. The concentration of changes into a small number of sites leads to more multiple hits and thus more amino acid replacements that are not observable and underestimation of divergence levels (see Supplementary Material Fig. S3; the nonlinearity of the relationship between *t*_*k*_^⁎^ and the inferred PAM distance also explains the gradient changes in the rate-heterogeneous AMP plot in Fig. 3b).

The considerable level of rate variation across sites needed to generate behavior similar to that observed by Benner *et al.* and Mitchison and Durbin could also be caused in part by variation in selective pressures such as those modeled by parameters of the M7 or M8 codon models for selection.^22^ Such study of the causes of rate heterogeneity is beyond the “proof-of-principle” approach used in this paper. Furthermore, our comparisons were limited, since the original data (and detailed results) of the above studies are not available anymore. However, the results of our simulation study using AMPs already strongly suggest that protein evolution will be most accurately modeled with codon rather than amino acid substitution models.

This recommendation is in accord with recent work on the use of codon-level models for molecular phylogenetics. Ren *et al.* study the utility of codon models for phylogenetic reconstruction and molecular dating.^44^ They report that codon models have good performance in both recent and deep divergences. Although their computational burden makes codon models currently infeasible for tree searching, Ren *et al.* recommend them for comparing predetermined candidate trees. In contrast, modeling protein sequence evolution on the amino acid level may introduce systematic error. The nature of protein-coding sequence evolution is such that time-homogeneous Markov modeling on the codon level seems reasonable, but this leads to time-dependent and non-Markov behavior on the amino acid level. It is increasingly feasible to use codon models where amino acid models have been used in the past, and our results overturn a long-standing claim that proteins evolve in a time-dependent manner and give further reasons why codon models may be preferable.

The following are the supplementary materials related to this article.

Supplementary materials
