# Supplementary material for: Markovian and Non-Markovian Protein Sequence Evolution: Aggregated Markov Process Models
Source: J Mol Biol. 2011 Aug 26;411(4-6):910–23. doi: 10.1016/j.jmb.2011.06.005 (PMC3157587; doi:10.1016/j.jmb.2011.06.005)
Supplement: Supplementary file 1 — Supplementary materials [file mmc1.pdf]

Supplement for: "Markovian and non-Markovian protein sequence evolution: aggregated Markov process models"

C. Kosiol and N. Goldman

Supplemental figures

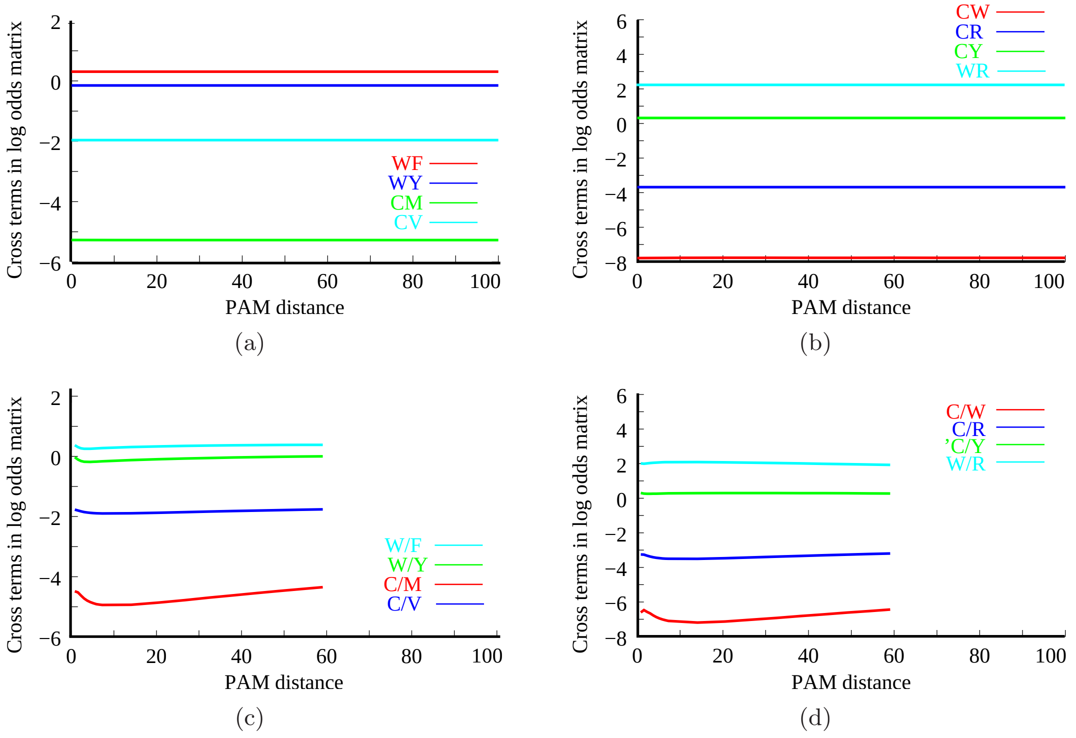

Figure S1: Time-homogeneous Markov processes on the amino acid level. Graphs of some off-diagonal elements of the 250 PAM log-odds matrix versus amino acid divergence times. The elements presented are identical to the the ones proposed by Benner *et al.*[1] Figs. S1(a) and (b) are estimated from data simulated under a simple time-homogeneous Markov process on the amino acid level; Figs. S1(c) and (d) under an amino acid model with rate variation among sites.

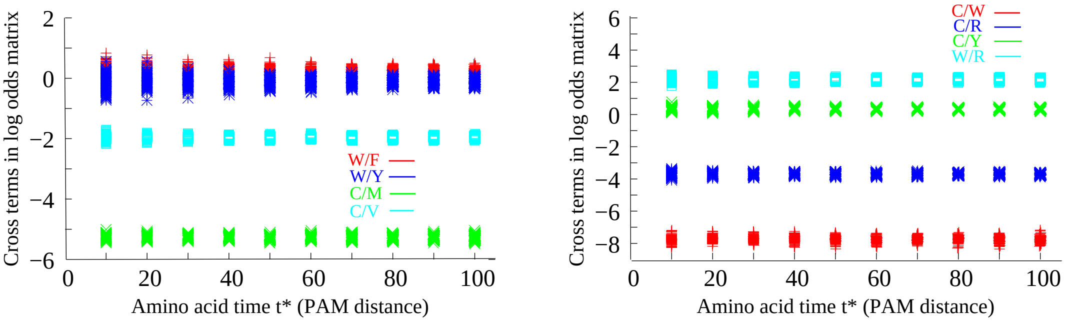

(a) (b)

Figure S2: Sampling variation of statistics reported by Bennner and colleagues. Benner *et al.*[1] included error bars in their plots illustrating observed time dependence of amino acid replacement. Although it is not possible to reproduce their data, we are able to infer from Benner *et al.*[1] that each data point was estimated from at least 200,000 amino acid pairwise alignments. Assuming a typical protein length of 150 amino acids, we sampled 100 data sets each comprising 200,000 amino acid pairwise alignments of length 150 amino acids, according to the Dayhoff time-homogeneous Markov model of amino acid replacement, at each divergence level used by Benner et al. Each simulated data set was subjected to the analyses of BCG, to enable us to see expected levels of inferential noise. Plots (a) and (b) show $L(250)$ cross terms and may be compared to the expectations shown in Fig. S1(a) and (b) and to BCG's results in Fig. 2(a) and (b). This indicates that BCG's observations cannot be explained as inferential noise about the expectations from a simple time-homogeneous Markov process on the amino acid level.

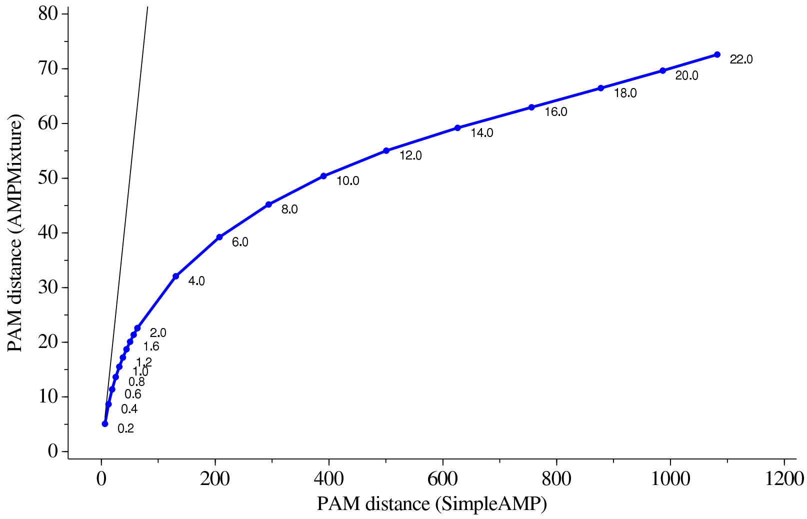

Figure S3: Systematic underestimation of PAM divergence for the AMP with 12 rate categories. The x-axis shows inferred PAM distance for data simulated with the simple AMP with no rate heterogeneity across sequence sites, and the y-axis shows inferred PAM distance for data simulated with the same numbers of codon changes using the AMP with 12 rate categories. These distances are referred to as t_k in the section 'Simulation methods' in the main text. Points on the line are labelled with the corresponding simulation times t_k^*, measured in codon changes per codon site. Notice how the inferred PAM distances for the AMP with 12 rate categories fall increasingly far below the thin black line $y = x$, indicating increasingly strong underestimation of amino acid sequence divergence level.

Supplemetal data

Substitution model used for the simulations

The parameter values of the codon model M0 (see Yang *et al.*[2] and eq. (3) in this article) were chosen typical for protein-coding cDNA:

```
\omega_M = 0.2
\kappa   = 2.5

61 codon frequencies \pi_j in alphabetic order AAA, AAC, ..., TTT

0.0140789      0.017353       0.0118961      0.0217186      0.0136648
0.0168427      0.0115462      0.0210798      0.00720507     0.00888067
0.00608801     0.0111148      0.0144101      0.0177613      0.012176
0.0222296      0.0119457      0.0147238      0.0100937      0.0184279
0.0115944      0.0142907      0.00979679     0.0178859      0.00611339
0.00753511     0.00516558     0.00943074     0.0122268      0.0150702
0.0103312      0.0188615      0.024958       0.0307622      0.0210886
0.0385011      0.024224       0.0298574      0.0204683      0.0373687
0.0127726      0.015743       0.0107924      0.0197035      0.0255453
0.031486       0.0215848      0.039407       0.0155126      0.0194151
0.0122155      0.0150563      0.0103216      0.0188441      0.00793878
0.00544231     0.00993596     0.0128818      0.0158776      0.0108846
0.0198719
```

References

[1] Benner, S.A., Cohen, M.A. & Gonnet, G.H. (1994). Amino acid substitution during functionally constrained divergent evolution of protein sequences. *Protein Eng*, **7**, 1323–1332.

[2] Yang, Z., Nielsen, R., Goldman, N. & Pedersen, A.M. (2000). Codon-substitution models for heterogeneous selection pressure at amino acid sites. *Genetics*, **155**, 431–449.
